# Consequences of the SARS-CoV-2 Infection on Anaerobic Performances in Young Elite Soccer Players

**DOI:** 10.3390/ijerph19116418

**Published:** 2022-05-25

**Authors:** Marc Dauty, Jérôme Grondin, Pauline Daley, Bastien Louguet, Pierre Menu, Alban Fouasson-Chailloux

**Affiliations:** 1 Nantes Université, Service de Médecine du Sport, CHU Nantes, 44093 Nantes, France; marc.dauty@chu-nantes.fr (M.D.); jerome.grondin@chu-nantes.fr (J.G.); pauline.daley@chu-nantes.fr (P.D.); bastien.louguet@chu-nantes.fr (B.L.); pierre.menu@chu-nantes.fr (P.M.); 2 Nantes Université, Service de Physique et Réadaptation Locomotrice et Respiratoire, CHU Nantes, 44093 Nantes, France; 3 Nantes Université, Inserm, UMR 1229, RMeS, Regenerative Medicine and Skeleton, ONIRIS, 44042 Nantes, France; 4 Institut Régional de Médecine du Sport, 44093 Nantes, France

**Keywords:** COVID-19, sport, adolescents, lockdown, soccer

## Abstract

The COVID-19 pandemic required local confinement measures reducing sport practice with possible consequences on the athletes’ performances. Furthermore, anaerobic detraining was underestimated and poorly known in adolescents. This article aimed to assess the effects of SARS-CoV-2 infection and 1-month COVID-19 confinement on jump testing in young elite soccer players despite a 1-month multimodal training program followed by a 1-month soccer retraining period. Thirty-one elite soccer players aged 14 were included; 16 were infected by the SARS-CoV-2 and compared with 15 non-infected elite soccer players before and after 1 month of COVID-19 confinement, and after 1 month of a soccer retraining period. Squat jumps (SJ), countermovement jumps with (CMJs) and without arm swinging (CMJ) and multiple consecutive jumps (stiffness) were used to explore the anaerobic performances. Analysis of variance for repeated measures was used to compare the positive and negative SARS-CoV-2 groups, taking into account the confinement period (low training) and the retraining soccer period. The jump tests were not altered in the positive SARS-CoV-2 group compared to the negative SARS-CoV-2 group after confinement (SJ: 31.6 ± 5.6 vs. 32.7 ± 3.7; CMJ: 34.1 ± 6.9 vs. 34.2 ± 2.6; CMJs: 38.6 ± 6.8 vs. 40.3 ± 3.9; stiffness: 28.5 ± 4.3 vs. 29.1 ± 3.7) and at 1 month of this period (SJ: 33.8 ± 5.5 vs. 36.2 ± 4.6; CMJ: 34.7 ± 5.5 vs. 36.4 ± 3.5; CMJs: 40.4 ± 6.7 vs. 42.7 ± 5.5; stiffness: 32.6 ± 4.7 vs. 34.0 ± 4.3). The SARS-CoV-2 infection had no consequence on anaerobic performances assessed by jump tests in adolescent soccer players. The adolescents’ growth could explain the absence of alteration of jump performances during the COVID-19 confinement. These results can be useful to manage the recovery of the anaerobic fitness after SARS-CoV-2 infection occurring in adolescent athletes.

## 1. Introduction

The COVID-19 pandemic has induced prolonged periods of sport detraining and competition suspension [1,2,3]. This suspension of activities had various effects on high-level or professional athletes [4,5,6], especially in terms of injury rate according to research from their countries, which might reflect differences concerning sports preparation after home confinement [5,6]. In professional soccer players, a 40-day COVID-19 lockdown has affected cardiovascular performances with a decrease of relative distance and maximal speed on the Yo-Yo test [7]. In the same way, the COVID-19 confinement has negatively affected cardiorespiratory fitness measured with the 20-m shuttle run (−0.5 mL/kg/min) in 14-year-old scholar boys and girls [8], or directly measured using laboratory instruments [9]. In elite adolescent soccer players, a reduction of 25% of the aerobic fitness was observed using a Yo-Yo test despite a home-training program [10].

From an anaerobic fitness point of view, the impact of COVID-19 confinement was debatable in professional soccer players. The vertical jumps were affected compared to a competitive period but not compared to the results after summer breaks [11]. In elite futsal adult players, no significant change was observed for countermovement jump (CMJ) height and horizontal jump distance, whereas sprint performances were affected [12]. The duration of the COVID-19 confinement may explain a decrease of performances in sprint and CMJ height compared to a traditional off-season [13]. However, no significant change was observed for hamstring eccentric strength and squat jump (SJ) or CMJ height [13]. Demir et al. have shown contradictory results for hamstring eccentric strength, and no change was shown for hip abductor and adductor strength [14]. In soccer referees, eccentric muscle strength evaluated by Nordic hamstring exercises decreased after the COVID-19 confinement, but a 4-week retraining was sufficient to solve the problem of muscle weakness [15]. In addition, home-based and group-based interventions in high-level female and male soccer players were efficient to maintain CMJ and SJ heights and sprints during the COVID-19 confinement [16,17,18].

In young athletes, the effects of the COVID-19 confinement in anaerobic fitness were also debatable. A 5-month confinement due to COVID-19 had a negative effect on scholar adolescents [19]. Jumping, sprinting and agility tests were impaired for both boys and girls [19]. However, the effect of a 3-week detraining period had no incidence on the CMJ in adolescents [20]. Therefore, the results on aerobic fitness detraining are controversial in such a population. In male professional football players, no significant difference before and after SARS-CoV-2 infection was found for CMJ, hip abductor and adductor muscle strength, and Nordic hamstring exercises [21]. However, no comparison has been performed between positive and negative SARS-CoV-2 groups. In elite sport adolescents, no data have been published on soccer players. Therefore, the aim of this study was to measure the consequences of the SARS-CoV-2 infection during a 1-month COVID-19 confinement on different jump tests.

## 2. Materials and Methods

### 2.1. Participants

All the young elite soccer players who played at the Pole Espoir of Saint Sébastien sur Loire, France, during the sports year 2020/2021, were eligible to participate in this study. Players had about 5 years of soccer experience. The written consents of the adolescents and of their parents were obtained to participate in the study. The study was approved by the Ethics Committee and the Research Direction of the Nantes University Hospital.

### 2.2. Symptoms of the SARS-CoV-2 Infection

All the adolescents were accommodated in a boarding school near the football center. None of the players were vaccinated. On 31 March 2021, the first young soccer player had fever, headache and cough. A RT-PCR (reverse transcription-polymerase chain reaction) was obtained the same day and confirmed a SARS-CoV-2 infection [22]. This adolescent was immediately isolated and went back home. Two days later, two other players had symptoms and a positive COVID-19 RT-PCR. Finally, 13 other young soccer players were ill with COVID-19 in a week (positive RT-PCR). All the other soccer players were tested by RT-PCR due to their contact with the ill players. Fifteen players have never tested positive for SARS-CoV-2 (two negative RT-PCR at 7 days of interval). The symptoms of SARS-CoV-2 infection were presented alone or in association: fatigue (five times), headaches (four times), fever (three times), body aches (three times) and cough (twice). Chills, loss of taste and smell, diarrhea and breathing difficulties were rarely presented (once for each symptom). Finally, no young soccer player needed to be hospitalized. At the same time, the third COVID-19 confinement began in France, from 3 April to 3 May 2021. The boarding school and the soccer training center were closed and stopped for 1 month.

### 2.3. Program of Exercises during Confinement and during the Retraining Period

Due to this COVID-19 confinement, a program of physical exercises was given to the negative SARS-CoV-2 group only (Table 1). For the positive SARS-CoV-2 group, rest was prescribed by a medical doctor for 10 days, and only a 20-min footing 3 times a week was recommended until the return to school, if all symptoms had disappeared [23]. All soccer players were individually followed once a week by trainers or school assistants using internet or phone calls [10]. After the COVID-19 confinement period, a progressive 1-month soccer retraining was resumed (Table 1) [24,25].

### 2.4. Jump Tests

**Figure 1:**
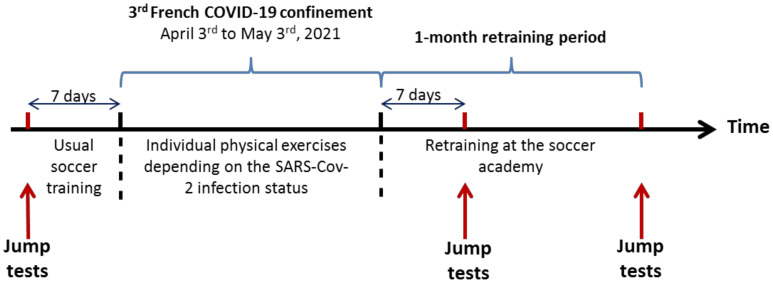
Experimental scheme of jumps assessment.

Jump tests were usually carried out once every 3 months during the soccer sport season and so, they happened in March 2021 (7 days before the confinement). These tests are used to measure the explosive strength of the lower limbs. Due to the COVID-19 confinement, jump tests were repeated 1 week and 1 month after the confinement period to evaluate the anaerobic consequences due to the SARS-CoV-2 infection and due to the COVID-19 confinement (Figure 1).

After a warm-up of 10 min jogging followed by 3 × 30 s standing on one leg, 10 squats, 10 front slots and 1 jumping try, all jump tests were realized using a photoelectric system (Optojump^®^ Microgate, Bolzano, Italy). Jump testing was always organized in the same order and supervised by the same trainer [26]. Ten minutes of active rest (walking and one jumping try) were taken between each jumping session.

1/Squat Jump (SJ):

From the squat position, knees flexed at 90 degrees, hands on hips, the subject had to jump vertically as high as possible, with his legs extended. The best of five tries (jump height) was selected for analysis. Thirty seconds of rest were granted between each try. The reliability of SJ with CMJ is considered high (Cronbach’s alpha = 0.97) [27].

2/Countermovement jumps with (CMJs) and without (CMJ) arm swinging movement:

From the erect position with trunk straight, knee in extension, the subject had to move downward until his knees were flexed approximately at 90 degrees and he had to make a maximal and explosive vertical jump, maintaining his legs extended. The best of the five tries (jump height) was selected for analysis. Thirty seconds were granted between every CMJ and CMJs. The hand position for the CMJ was on hips and arm swinging movement was authorized for the CMJs. The test-retest reliability separated by 1 week is excellent (intraclass coefficient: 0.982–0.989) with a low random errors of +/−2.8 cm [28].

3/Stiffness test:

This test was performed with an Optojump^®^ Microgate (Bolzano, Italy). This device is an optical measurement system which measures flight during the performance of a series of jumps with an accuracy of 1/1000 of a second. Stiffness is a quantitative measure of the elastic properties of the body and it determines the ability to accumulate potential elastic energy [29]. Muscle tension is a factor regulating the stiffness of the support limb during jumps [30]. This test measured the reactive force during seven vertical jumps realized twice (1 min rest), hands on hips, with straight knees. The elevation of the center of gravity was measured for all the seven vertical jumps. Only the mean elevation of the body gravity center of the best series of seven jumps was selected for analysis. The test-retest reliability separated by 1 week is high (intraclass coefficient: 0.82–0.86) [31].

### 2.5. Statistical Analysis

The statistical analysis was performed using SPSS 23.0^®^ software (Armonk, NY, USA). Quantitative variables were given in mean and standard deviations. Variance normality was tested by the Kolmogorov–Smirnov test. At baseline, the positive and negative SARS-CoV-2 groups were compared with a t-test after variance comparison by Levene test. The COVID-19 confinement effects and the retraining soccer period were measured by comparing the jump test variable using an analysis of variance for repeated measures (3 times × 2 groups). The assumption of sphericity was assessed and corrected using the epsilon of Greenhouse–Geisser. Paired-comparisons were performed with Bonferroni test. Effect sizes were assessed by partial eta squared η^2^, which were defined as trivial, small, moderate and large for values η^2^ ≤ 0.1, ≤0.3, ≤0.5 and >0.5, respectively [32]. The alpha level of statistical significance was set at *p* < 0.05.

## 3. Results

Thirty-two young soccer players aged 14 were eligible. One adolescent was excluded due to an ankle injury. Sixteen of them were infected by the SARS-CoV-2. Thirty-one players performed anaerobic tests (jump tests) 1 week before this third COVID-19 confinement. The positive and negative groups for the virus were not different for anthropometric and jump parameters before the COVID-19 confinement (Table 2 and Table 3). No effect of the SARS-CoV-2 infection was observed, but only a time effect according to the types of jump tests (Table 3).

**Figure 2:**
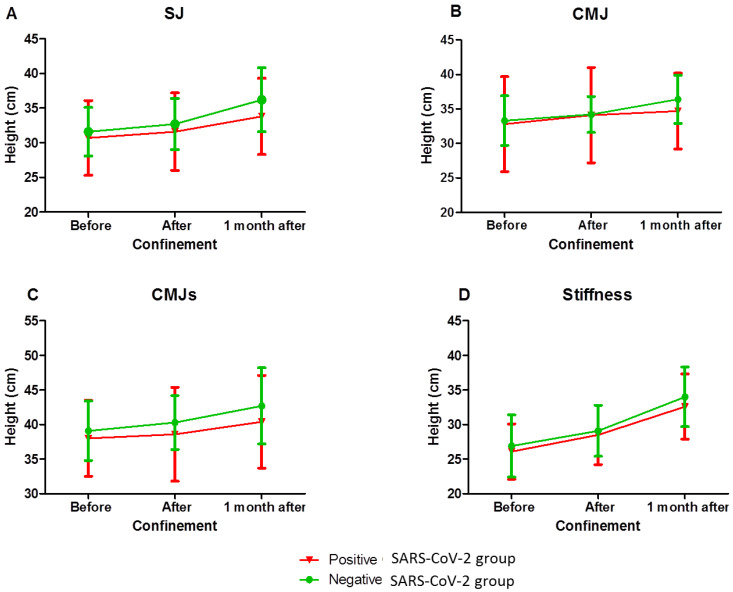
Jump parameter evolution before, after and at 1 month of the COVID-19 confinement in players who had SARS-CoV-2 infection (red line) or not (green line). (**A**) SJ parameters (cm) evolution; (**B**) CMJ parameters (cm) evolution; (**C**) CMJs parameters (cm) evolution; (**D**) stiffness parameters (cm) evolution.

The SJ, CMJ, CMJs and stiffness parameters increased in the same way (trivial size effect: η^2^ ≤ 0.1) for the positive and negative SARS-CoV-2 groups (Figure 2). The time effect was not significant for the CMJ (Figure 2A). A significant improvement for SJ and for CMJs parameters was observed only after COVID-19 confinement and 1 month later (mean difference: D = 2.87; *p* < 0.001 and D = 2.19; *p* < 0.01, respectively) (Figure 2B,C). A significant improvement for stiffness parameters was observed before and after COVID-19 confinement (D = 2.28; *p* < 0.01), and after COVID-19 confinement and 1 month later (D = 4.5; *p* < 0.0001) (Figure 2D).

## 4. Discussion

To our knowledge, it is the first demonstration of the absence of alteration of jumping performances (SJ, CMJ, CMJs and stiffness) by the SARS-CoV-2 infection in adolescent elite soccer players. A previous study showed similar results for the CMJ in adult professional soccer players, although no comparison had been made with a control group [21]. The mean height of CMJ was 37.4 cm 1 week before COVID-19 and was stable at 2, 4, 6 and 8 weeks post-COVID-19 (39.8 to 41.7 cm) without any significant change [21]. Nevertheless, infection usually leads to a decrease of muscle strength, particularly during the symptomatic period. Viral infection also leads to a decrease of the muscle protein content, which correlates to a decline of the muscle strength [33,34]. It might take up 2 weeks for the muscle protein to be restored [33]. In addition, in the case of myalgia, which is a common symptom of SARS-CoV-2 infection, the motor coordination could be impaired due to a possible disturbance in neuromuscular transmission [33]. Muscle damage could be due to an increase of inflammatory cytokines, explained by the infection of SARS-CoV-2 which targets cells using different receptors such as the TMPRSS2 (type 2 transmembrane serine protease receptors) [35]. However, because soccer adolescents were advised not to train during their infection (rest for 10 days and only aerobic training afterwards), this could explain the absence of alteration of the jumping performances measured 1 month after infection, due to the full infection recovery. Indeed, the return to exercise for patients recovering from COVID-19 should be guided by the disappearance of the symptoms [35].

The 1-month COVID-19 confinement had also no consequences on the jumping performances. On the contrary, the jumping height reached had increased especially during the retraining period. The duration of the confinement was perhaps not enough to significantly decrease the anaerobic performances. Mujika et al. also showed in 2000 that only 7–12% of strength was lost during 12 weeks of detraining in adults (maximum of 1% per week) [36]. Several months may be needed to notice a significant loss of anaerobic capacity. However, an improvement of jumping performances was shown during the COVID-19 confinement in our study. This result can been explained by the dependence of squat and countermovement jump height to the pubertal status and the level of sport [37,38]. Before the COVID-19 confinement, the SJ and CMJ heights of our soccer player population aged 14 were similar to the heights of the soccer populations of the same age studied by different authors [37,39,40,41]. In a younger population aged 12 (pre-pubertal period), the SJ and CMJ height performances were lower (20 and 21.5 cm, respectively), and they were higher after the pubertal period (36 and 37.5 cm, respectively) [42]. In the same way, Cunha et al. have shown an increase of jump height depending on the pubertal status for the SJ (32 to 38.6 cm) and CMJ (34.7 to 38.6 cm) [37].

The jump performances also depend on the level of sport [38]. Indeed, Murtagh et al. have recently pointed out that elite soccer players had higher jump performances compared to a control group of adolescents from 13 to 17 years of age for the CMJ [43]. In addition, a strength training program was effective to improve CMJ height performances in soccer players aged 15.2 [44]. Therefore, our post-confinement retraining period could explain the improvement of jump performances. This retraining was more effective compared to the self-training period observed during the COVID-19 confinement.

### Limitations

The positive and negative SARS-CoV-2 groups were rather small to obtain reliable results but our group corresponded to a homogenous population of elite adolescent soccer players. Yet, this population seems not highly sensitive to the most harmful effects of SARS-CoV-2. Other anaerobic testing such as sprinting or limb strength tests could have been used to better explore the anaerobic capacities. Unfortunately, no sprinting tests were carried out just before the COVID-19 confinement because it was not possible to know the exact date of the confinement or if some adolescents had been infected or not by SARS-CoV-2. Moreover, the effects of the SARS-CoV-2 infection and a 1-month COVID-19 confinement may be different in an untrained population. Finally, as the sample does not present complications or long periods of confinement, it is difficult to predict the possible harmful effects of the virus.

## 5. Conclusions

Anaerobic performances measured using squat jumps, countermovement jumps and stiffness seemed not to be altered by the SARS-CoV-2 infection in adolescent elite soccer players. In the same way, a 1-month COVID-19 confinement appeared insufficient to alter the jump performances in these growing soccer players. These results could be useful to manage the recovery of anaerobic fitness after a viral infection occurring in adolescent athletes.

## Figures and Tables

**Table 1:** Typical weeks of training during the 1-month COVID-19 confinement for the negative SARS-CoV-2 group and the 1-month retraining soccer period for the two groups.

	Exercises during COVID-19 Confinement for Negative SARS-CoV-2 Group	Soccer Retraining after COVID-19 Confinement for the Two Groups
Monday	Aerobic running (35–40 min)	Aerobic recovery (20–45 min)
Tuesday	Long aerobic interval (3–5 × 3 min)	Aerobic power (30–75 min)
Wednesday	Short aerobic interval (30 s/30 s during 10 min × 1–2)	Anaerobic power (30–90 min)
Thursday	Pyramidal speed running (5–10–15–20–25–20–15–10–5 m × 1–3)	Speed and sprint (15–60 min)
Friday	Strengthening exercises	Intensity (30–45 min)
Saturday	Short interval training (15 s/15 s during 5–10 min and 5 s/25 s during 3–6 min)	Match in competition
Sunday	Rest	Rest

The improvement of the duration and series is mentioned in the bracket from the minimum (first week) to maximum (last week).

**Table 2:** Comparison of anthropometric parameters between positive and negative SARS-CoV-2 groups before confinement.

	Positive SARS-CoV-2 Group (*n* = 16)	Negative SARS-CoV-2 Group (*n* = 15)	*p*
Weight (kg)	56.2 ± 9.3	55.1 ± 9.1	0.74
Height (cm)	171.0 ± 9.0	169.0 ± 9.0	0.68
Body Mass Index (kg/m^2^)	19.0 ± 1.4	18.9 ± 1.3	0.91

**Table 3:** Evolution of jump parameters in the positive and the negative SARS-CoV-2 groups (ANOVA between groups at each time-point: 3 time-points × 2 groups).

	Positive SARS-CoV-2 Group (*n* = 16)			Negative SARS-CoV-2 Group (*n* = 15)			F	*p*	η^2^
Confinement	Before	After	1 Month	Before	After	1 Month			
SJ (cm)	30.7 ± 5.4	31.6 ± 5.6	33.8 ± 5.5	31.6 ± 3.5	32.7± 3.7	36.2 ± 4.6	1.23	0.30	0.06
CMJ (cm)	32.8 ± 6.9	34.1 ± 6.9	34.7 ± 5.5	33.3 ± 3.6	34.2 ± 2.6	36.4 ± 3.5	0.98	0.38	0.05
CMJs (cm)	38.0 ± 5.5	38.6 ± 6.8	40.4 ± 6.7	39.1 ± 4.3	40.3 ± 3.9	42.7 ± 5.5	0.41	0.65	0.02
Stiffness (cm)	26.1 ± 4.0	28.5 ± 4.3	32.6 ± 4.7	26.9 ± 4.5	29.1 ± 3.7	34.0 ± 4.3	0.19	0.80	0.01

SJ: squat jump; CMJs: countermovement jumps with arm swinging movement; (CMJ): countermovement jumps without arm swinging movement.

## Data Availability

The data presented in this study are available on request from the corresponding author. The data are not publicly available due to ethical reasons.
